# Molecular characterization of *Brucella* species from Zimbabwe

**DOI:** 10.1371/journal.pntd.0007311

**Published:** 2019-05-20

**Authors:** Maphuti Betty Ledwaba, Calvin Gomo, Kgaugelo Edward Lekota, Philippe Le Flèche, Ayesha Hassim, Gilles Vergnaud, Henriette van Heerden

**Affiliations:** 1 Department of Veterinary Tropical Diseases, University of Pretoria, Pretoria, South Africa; 2 Central Veterinary Laboratory (CVL), Harare, Zimbabwe; 3 Chinhoyi University of Technology, Department of Animal Production and Technology, Harare, Zimbabwe; 4 Institute for Integrative Biology of the Cell (I2BC), CEA, CNRS, Univ. Paris-Sud, Université Paris-Saclay, Gif-sur-Yvette, France; Instituto Butantan, BRAZIL

## Abstract

*Brucella abortus* and *B*. *melitensis* have been reported in several studies in animals in Zimbabwe but the extent of the disease remains poorly known. Thus, characterizing the circulating strains is a critical first step in understanding brucellosis in the country. In this study we used an array of molecular assays including AMOS-PCR, Bruce-ladder, multiple locus variable number tandem repeats analysis (MLVA) and single nucleotide polymorphisms from whole genome sequencing (WGS-SNP) to characterize *Brucella* isolates to the species, biovar, and individual strain level. Sixteen *Brucella* strains isolated in Zimbabwe at the Central Veterinary laboratory from various hosts were characterized using all or some of these assays. The strains were identified as *B*. *ovis*, *B*. *abortus*, *B*. *canis* and *B*. *suis*, with *B*. *canis* being the first report of this species in Zimbabwe. Zimbabwean strains identified as *B*. *suis* and *B*. *abortus* were further characterized with whole genome sequencing and were closely related to reference strains 1330 and 86/8/59, respectively. We demonstrate the range of different tests that can be performed from simple assays that can be run in laboratories lacking sophisticated instrumentation to whole genome analyses that currently require substantial expertise and infrastructure often not available in the developing world.

## Author summary

Brucellosis is endemic in Zimbabwe. This article describes the use of various assays such as AMOS, Bruce-ladder, MLVA, and whole genome sequencing to characterize *Brucella* species isolated from different animals in Zimbabwe. Choice of which assays to use in the laboratory is generally done considering reproducibility, robustness, expertise and affordability in a given setting. As evidenced in this study, most laboratories in Africa lack resources especially finances, equipments and expertise to perform necessary tests for diagnosis and identification of specific pathogens. The study shows that the differentiation of species can be correctly concluded from the analysis with AMOS, Bruce-ladder and MLVA16 assays. Furthermore, MLVA16 can be used as an epidemiological tool and traceback of outbreaks. These PCR assays can therefore add to the control and eradication of brucellosis, since the *Brucella* species (*B*. *ovis*, *B*. *abortus*, *B*. *suis* and *B*. *canis*) existing in Zimbabwe could be identified and characterized.

## Introduction

Brucellosis is a worldwide infectious disease affecting a wide range of domestic and wildlife animals and humans [1]. Brucellosis is caused by species in the genus *Brucella*, which consists of six classic species, *Brucella abortus*, *B*. *melitensis*, *B*. *suis*, *B*. *ovis*, *B*. *canis*, and *B*. *neotomae* [2]. Recently, the genus has expanded to include *B*. *ceti* and *B*. *pinnipedialis* from marine animals [3], *B*. *microti* from the common voles (*Microtus arvalis)* [4] and red foxes [5], *B*. *inopinata* isolated from a human breast implant [6], *B*. *papionis* from baboons (*Papio* spp.), *B*. *vulpis* from red foxes (*Vulpes vulpes*) and novel *Brucella* spp. in amphibians and fish [7, 8, 9, 10].

In African countries, brucellosis is reported to be a serious threat; although, under-reported due to a limited number of studies conducted and the lack of epidemiological evidence [11]. *B*. *abortus* and *B*. *melitensis* have been reported frequently when livestock have been tested. However, there is limited information on the prevalence of brucellosis in small ruminants as compared to cattle [12]. In Zimbabwe, only *B*. *abortus* and *B*. *melitensis* have been reported to cause brucellosis in animals [13], with *B*. *abortus* biovar (bv.) 1 and to a lesser extent *B*. *abortus* bv. 2 reported to be the most prominent cause of bovine brucellosis [13]. However, reports might be biased because investigations/ testing targeted mostly bovine rather than other species. The authors used biotyping and AMOS-PCR to identify *Brucella* isolates from commercial and communal cattle farms in Zimbabwe and also reported a single *B*. *melitensis* bv. 1 isolate from a goat. Brucellosis has been demonstrated by serology to be present in Zimbabwean wildlife including African buffalo, eland, zebra, giraffe and impala [14] as well as in domestic dogs [15]. *B*. *abortus* bv. 1 was isolated from waterbuck (*Kobus ellipsiprymnus*) and eland (*Taurotragus oryx*) [16]. This complicates the control of the disease since the animals in areas bordering the National parks interact with wildlife and it is almost impossible to vaccinate wildlife. Bovine brucellosis is endemic in the country in most regions with high sero-prevalence of up to 53% reported in commercial herds as compared to 16% from small-scale farmers in Zimbabwe [17, 18].

General classification of *Brucella* species and biovars is still based on phenotypic characteristics, with minimal standards previously defined [19] well before the development of modern genomics and the discovery of new *Brucella* species. Biotyping is time-consuming and often difficult to interpret due to limited standardization of the typing reagents [20]. Moreover, the efficacy of biotyping is moderate and since it includes the manipulation of the live agent, it poses a biosafety and public health risks of laboratory infections to the personnel involved [20]. Initial assays based on DNA analysis by PCR amplification were genus-specific and not sufficient to assist brucellosis control programs [21, 22] in the endemic regions of Zimbabwe. Most programs for brucellosis control employ genus-specific serology tests which are confirmed by species-specific culturing since the associated regulatory methods are species dependent [23]. AMOS-PCR is a multiplex PCR assay that differentiates *B*. *abortus* bv. 1, 2 and 4, *B*. *melitensis*, *B*. *ovis*, *B*. *suis* bv. 1, *B*. *abortus* vaccine strains S19 and RB51 based on the genetic element *IS711* [24, 25]. AMOS and Bruce-ladder multiplex PCRs use species- and strain-specific genetic differences to distinguish among *Brucella* species [26, 27]. The initial Bruce-ladder assay identifies almost all *Brucella* species including the vaccine strains *B*. *abortus* S19, RB51 and *B*. *melitensis* Rev1 but will occasionally incorrectly identify some *B*. *canis* strains as *B*. *suis* [27]. The original Bruce-ladder assay had limited utility for distinguishing the more recently described species such as *B*. *ceti*, *B*. *pinnipedialis*, *B*. *microti*, and *B*. *inopinata;* but was later updated [28; 29, 30]. Multi-locus variable number tandem repeats (VNTR) assays (MLVA) is a genetic approach with high discriminatory power in the *Brucella* genus, clearly identifying species and providing fine-scale resolution among isolates [31, 32]. The most commonly used MLVA scheme consists of 16 VNTR markers, including eight moderately variable minisatellites (panel 1) and eight highly polymorphic microsatellites (panel 2A and 2B) [20, 33] that has the capacity to distinguish *Brucella* species and their biovars. Accurate discrimination between species and biovars achieved with the high resolution MLVA is necessary to determine the source, origin and geographical spread of infection [34]. Finally, characterisation of the genome of *Brucella* species with whole genome sequencing (WGS) provides the ultimate genetic resolution and can enable the determination of other features such as virulence factors [35]. The availability of whole genome sequences covering *B*. *melitensis* [36], *B*. *suis* [37], and *B*. *abortus* [38] has contributed to our understanding of the pathogenicity and diagnosis of brucellosis [35, 36]. WGS combined with single nucleotide polymorphism (SNP) analysis provides greater resolution and fine-scale differentiation of *Brucella* species [39, 40, 41] that cannot be obtained with multiplex PCR assays or MLVA.

*Brucella abortus* bv.1 is the most frequently isolated species in the cattle industry in Zimbabwe, with *B*. *abortus* bv.2 occasionally detected [13]. However, the control program is compulsory for commercial farming but is only optional for communal cattle production systems so may be missing most cases of brucellosis [14]. Various strains have been isolated from samples collected between 1990 and 2009 from various host animals throughout the country at the Central Veterinary Laboratory (CVL) in Zimbabwe, with only some of the isolates previously identified to the species level using biotyping. The aim of this study was to characterize these *Brucella* strains using AMOS-PCR, Bruce-ladder and MLVA, to evaluate genotyping approaches and develop a toolkit to support a nation-wide eradication program at a sustainable cost. Finally, based on the data obtained with the abovementioned techniques, three isolates were further characterized with WGS [42].

## Methods

### Ethics statement

All experimental protocols were approved by the Animal Experiments and Ethics Committee of the University of Pretoria (V096-15 AEC Approval) and the Section 20 approval obtained from DAFF (SDAH-Epidem 15012613530_ Section 20) for the use of animals and animal products.

### Bacterial strains and bacteriology

Sixteen *Brucella* strains (Table 1) were isolated at CVL from samples of domestic animals collected and isolated isolated between 1990 and 2009 in Zimbabwe and used to evaluate the feasibility and the need of large scale surveillance in the country. At the time of the study (2011–2013) there was no surveillance going on, the study isolates were obtained from farms/clients samples submitted to CVL for routine screening. They were characterized as *Brucella* by bacteriological methods (urease, catalase, oxidase, H_2_S, indole and sensitivity to dyes (thionin and basic fuchsin)) as indicated by previously [43]. Due to financial constraints, it was not possible for the laboratory to buy PCR reagents at that time; thus, only 7 of the 16 cultures were further classified to species level with the available reagents (S1 Table) according to standard bacteriological methods (excluding the phage lysis test) [43].

**Table 1:** List of field strains isolated from samples collected and isolated from 1990–2009 in Zimbabwe and the reference strains used in the study as well as their spp. identity, hosts and place of origin.

Strain number	Alias#	Species	Host (source)	Country^‡^
ZW002	2	*Brucella*	Sheep	Gwanda, ZW
ZW005*	5	*B*. *ovis*	Sheep	Insiza, ZW
ZW248	248	*Brucella*	Cow	Mazowe, ZW
ZW283*	283	*B*. *abortus*	Cow	Gwanda, ZW
ZW011*	11	*B*. *suis*	Pig	Shamwa, ZW
ZW040	40	*B*. *suis*	Cattle (testicles)	Bindura, ZW
ZW043	43	*B*. *suis*	Cattle	Chiredzi, ZW
ZW045	45	*B*. *suis*	Cattle (testis)	Bindura, ZW
ZW046	46	*B*. *suis*	Cattle	Norton, ZW
ZW047	47	*B*. *suis*	Cattle (milk)	Zimbabwe
ZW048	48	*B*. *suis*	unknown	Zimbabwe
ZW201*	201	*B*. *suis*	Pig	Norton, ZW
ZW053	53	*B*. *abortus*	Cattle	Matabeland, ZW
ZW323*	323	*B*. *abortus*	Cattle	Harare, ZW
ZW100*	100	*B*. *canis*	Dog	Harare, Highlands, ZW
ZW377*	377	*B*. *canis*	Dog	Harare, ZW
BCCN R7^#^	REF 292	*B*. *abortus* bv 4	Cattle	England
BCCN R6^#^	REF Tulya	*B*. *abortus* bv 3	Human	Uganda
BCCN R5^#^	REF 86/8/59	*B*. *abortus* bv 2	Cattle	England
BCCN R4^#^	REF 544	*B*. *abortus* bv 1	Cattle	England
BCCN R3^#^	REF Ether	*B*. *melitensis* bv 3	Goat	Italy
BCCN R22^#^	REF Reo 198	*B*. *ovis*	Sheep	USA
BCCN R21^#^	REF 513	*B*. *suis* bv 5	Wild rodent	Former USSR
BCCN R2^#^	REF 63/9	*B*. *melitensis* bv 2	Goat	Turkey
BCCN R18^#^	REF RM 6/66	*B*. *canis*	Dog	USA
BCCN R17^#^	REF BOW 63/290	*B*. *ovis*	Sheep	Australia
BCCN R15^#^	REF 40	*B*. *suis* bv 4	Reindeer	Former USSR
BCCN R14^#^	REF 686	*B*. *suis* bv 3	Swine	USA
BCCN R13^#^	REF Thomsen	*B*. *suis* bv 2	Swine	Denmark
BCCN R12^#^	REF 1330	*B*. *suis* bv 1	Swine	USA
BCCN R11^#^	REF C68	*B*. *abortus* bv 9	Cattle	England
BCCN R1^#^	ATTC 23456	*B*. *melitensis*	Goat	USA
BCCN R9^#^	REF 870	*B*. *abortus bv6*	Cattle	Africa
BCCN R8^#^	REF B3196	*B*. *abortus bv5*	Cattle	England
BCCN R16^#^	REF 5K33	*B*. *neotomae*	Desert rat	USA

*Identified using growth characteristics and biochemical profiles

# REF: reference DNA obtained from BCCN (*Brucella* Culture Collection Nouzilly, France).

^**‡**^ ZW: strain isolated from Zimbabwe

DNAs from 17 reference strains obtained from National and OIE/FAO Animal Brucellosis Reference Laboratory in France were included as controls for PCR assays (Table 1). Genotyping information of *Brucella* strains from previous studies [20, 31, 32, 44] that were used in MLVA in this study can be accessed from MLVA database [45].

### DNA preparation

DNA was extracted from each strain grown on *Brucella* selective media and blood agar using Qiagen DNA mini kit (Qiagen) at CVL in Zimbabwe and quantified with BioTek Take3 Micro-Volume Plate used in BioTek Microplate reader using the Gen5 pre-programmed quantification protocol at the University of Pretoria, South Africa. The study controls were amplified with Genomiphi DNA Amplification Kit (GE Healthcare Life Sciences AEC-Amersham) to increase their quantity.

### AMOS-PCR and Bruce-ladder

AMOS-PCR was done as described previously [25, 26]. The PCR mixture contained 1X MyTaq mix (Bioline), a combination of five primer sets specific for *B*. *abortus*, *B*. *melitensis*, *B*. *ovis*, *B*. *suis* (0.2 μM) and *IS711* (1 μM), respectively, and 10 ng DNA per 25 μl reaction. The PCR conditions consisted of an initial denaturation at 95°C for three minutes followed by 35 cycles of 95°C for one minute, 55.5°C for two minutes and 72°C for two minutes.

Bruce-ladder PCR was also done as described previously [27]. PCR reactions (25 μl) composed of 1X MyTaq mix (Bioline), 0.4μM of each primer of the eight primer pairs and 10ng template DNA. PCR conditions consisted of initial denaturation at 95°C for three minutes, followed by 25 cycles at 95°C for 30 sec, 64°C for 45 sec and 72°C for three minutes and a final extension of 72°C for five minutes on an ABI 2720 Thermal Cycler (Applied Biosystems).

To confirm the identity of strains identified as *B*. *suis* and *B*. *canis* with Bruce-ladder, the previously described Suis-ladder multiplex PCR assay [46] was used.

PCR products were separated by gel electrophoresis on a 1.5% agarose gel subsequently stained with ethidium bromide and photographed under UV light.

### MLVA

MLVA16 was performed as previously described [20, 31]. The 16 locus set was divided in three groups namely panel 1 (bruce06, bruce08, bruce11, bruce12, bruce42, bruce43, bruce45 and bruce55), panel 2A (bruce18, bruce19, bruce21) and panel 2B (bruce04, bruce07, bruce09, bruce16 and bruce30). PCR was performed in 15 μl reactions containing 3–15 ng of DNA template, 1X PCR buffer (Promega), 200 μM of each deoxynucleotide triphosphate, 0.5 μM of each flanking primer [20,31] and 1U Go*Taq* Hotstart polymerase (Promega). The PCR conditions included an initial denaturation step of 96°C for five minutes, followed by 30 cycles of 96°C for 30 seconds, 60°C for 30 seconds, extension at 72°C for one minute, followed by a final extension step of 72°C for 5 minutes. The PCR reaction products (5 μl) were separated on agarose gels in 1x TAE buffer using electrophoresis until the bromophenol blue has run for 20 cm on the agarose gel. The 16M *B*. *melitensis* reference strain was included as a control since each VNTR locus size is known. *Brucella* reference strains that have already been characterized using the MLVA16 markers panel 1, panel 2A and panel 2B were included to ensure accurate evaluation of field strain genotypes. For Panel 1 VNTRs, 2% agarose gel was used with GeneRuler 100 bp plus DNA ladder (Thermo Scientific). For panel 2 VNTRs, 3% standard agarose gel and low molecular weight DNA ladder 766–25 bp (New England Biolabs) were used. The ethidium bromide stained gels were visualized by UV light. Genotype was scored by visual analysis of the gel images or BioNumerics software version 6.6 (Applied-Maths).

### Data analysis

Band size estimates were converted to repeat units following the published allele numbering system version 3.6 [45] (S1 Table). MLVA data were analysed as a character data set within BioNumerics software (version 6.6) (Applied Maths). Clustering analysis was performed using the categorical coefficient and UPGMA (unweighted pair group method using arithmetic averages). A different weight was given to the markers depending on their panel: Panel 1 markers were assigned an individual weight of 2 (total weight for panel 1: 16), panel 2A markers a weight of 1 (total weight for panel 2A: 3), and markers of panel 2B a weight of 0.2 (total weight for panel 2B: 1) [20]. The MLVA16 results were compared with MLVA16 published data of *Brucella* reference and other strains [20, 31, 44] (S1 Table). Minimum spanning tree (MST) analysis was performed using MLVA8 (panel 1) in BioNumerics as well.

### Whole genome sequencing (WGS) and single nucleotide polymorphism (SNP) analyses

Zimbabwean *B*. *suis* strains ZW043 (GenBank accession CP009094.1 and CP009095.1) and ZW046 (GenBank accession CP009096.1 and CP009097.1) and *B*. *abortus* strain ZW053 (GenBank CP009098.1 and CP009099.1) [47] were selected for WGS since these strains were isolated from cattle in different regions of Zimbabwe and represented different MLVA genotype subclades. In addition, 23 *B*. *abortus* and 17 *B*. *suis* complete genomes were retrieved from GenBank and used for comparison and phylogenetic analyses (S2 Table). Sequenced reads from *B*. *abortus* and *B*. *suis* strains were aligned to *B*. *abortus* str. 9–941 (Accession no: NC_006932.1, NC_006933.1) and *B*. *suis* 1330 (Accession no: NC_017251, NC_017250) respectively, using Burrows-Wheeler Aligner (BWA) [48]. SAMtools [49] was used to sort and index the aligned reads of *Brucella* genomes. Sequence reads of the complete and draft *Brucella* genomes were simulated using SAMtools [49]. Picard-tools (http://picard.sourceforge.net/) were used to mark duplicate reads and to build binary index of the samples. Repeated regions of the *Brucella* sequenced reads were excluded from this analysis. For variant detection, Unified Genotyper method in GATK [50] was used to call for SNPs. Variant filtration and selection of SNPs was achieved using GATK. SNPs positioning sets were deducted from the aligned genomes using molecular evolutionary genetics analysis (MEGA) tool version 6 [51]. Only SNP positions that could be called in all genome sequences were used (core genome analysis) for phylogenetic analysis. A phylogenetic tree was constructed using (MEGA) tool version 6 [51] from the coreSNPs of the *Brucella* genomes. The trees were generated using maximum likelihood method with 500 bootstrap replicates.

## Results

### Bacteriology

All *Brucella* spp. strains from Zimbabwe were non-motile, gram-negative coccobacilli, positive for modified Ziehl-Neelsen stain, negative for indole production, and oxidase and catalase production positive. Only a few of the strains (S1 Table) were further characterized using growth characteristics and biochemical profiles (phage lysis was not determined).

### AMOS-PCR and Bruce-ladder

**Fig 1:**
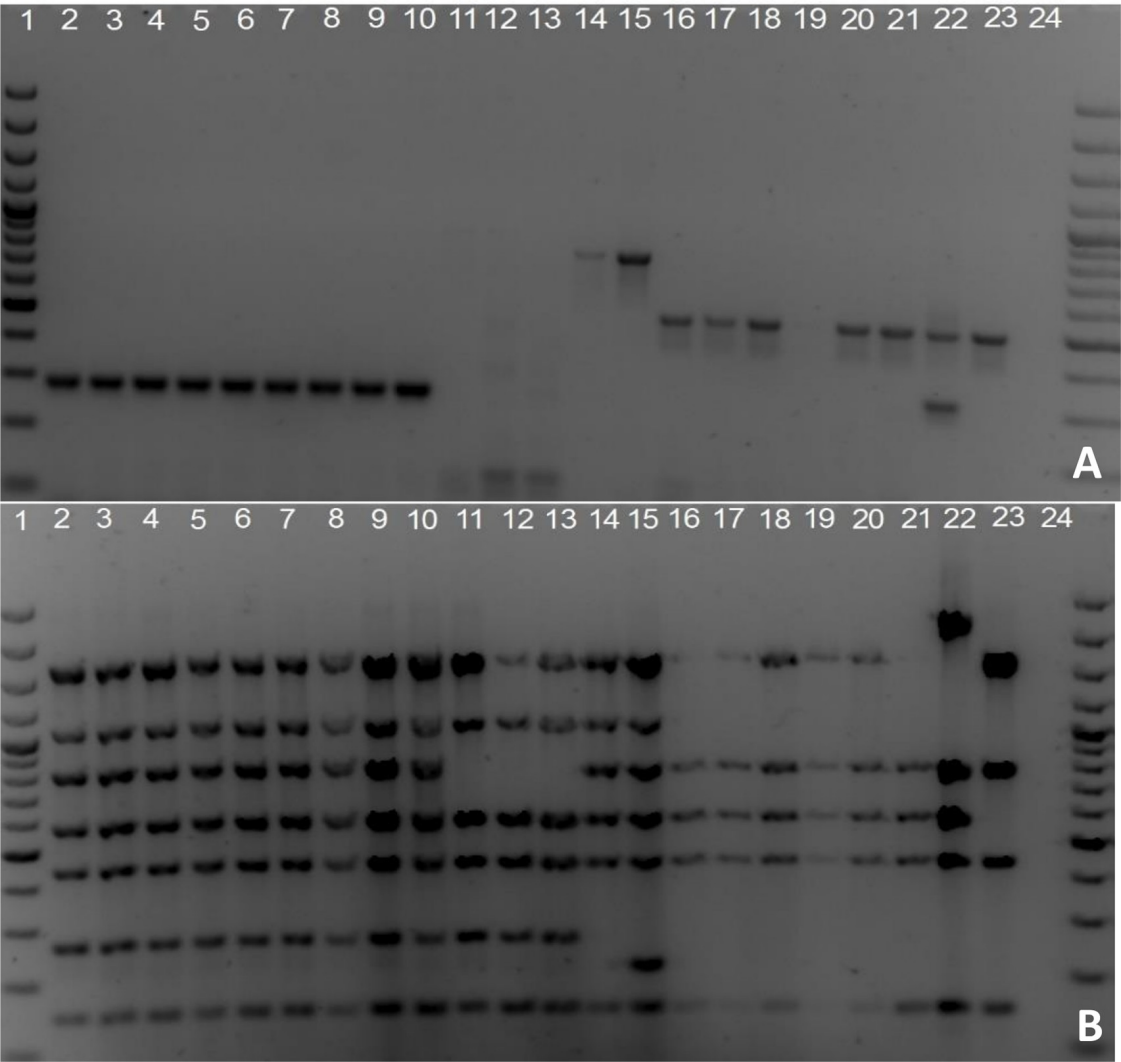
**Multiplex PCR assays (A) AMOS-PCR and (B) Bruce-ladder differentiation of Zimbabwean and reference *Brucella* strains**. Lane 2–10: *B*. *suis* bv. 1 (1330, BCCN R12), ZW011, ZW040, ZW043, ZW045, ZW046, ZW047, ZW048, ZW201; lane 11–13: *B*. *canis* (RM 6/66, BCCN R17), ZW100, ZW377; lane 14–15: *B*. *melitensis* bv. 1 (16M, BCCN R1), *B*. *melitensis* rev 1; lane 16–23: *B*. *abortus* bv. 1 (544, BCCN R4), ZW053, ZW086, ZW248, ZW283, ZW323, *B*. *abortus* RB51, *B*. *abortus* S19; lane 24 negative (water) control; lane 1 Fermentas 100 bp marker plus.

All strains except ZW100 and ZW377 were successfully genotyped using AMOS-PCR (Fig 1A). ZW002 and ZW005 were identified as *B*. *ovis*, ZW011, ZW040, ZW043, ZW045-048, ZW201 as *B*. *suis* and ZW053, ZW248, ZW283 and ZW323 as *B*. *abortus* (Fig 1A and Table 2).

**Table 2:** Summary table indicating the results obtained with AMOS, Bruce-ladder and MLVA testing the isolates from Zimbabwe.

Strain No.	Alt. number	Host	AMOS & Bruce-ladder	Suis-ladder	Genotype		
					MLVA		
					8	11	16
ZW011	11	Pig	*B*. *suis* bv. 1	*B*. *suis* bv. 1	6		NEW_1
ZW040	40	Cattle	*B*. *suis* bv. 1	*B*. *suis* bv. 1	6	33	NEW_2
ZW043	43	Cattle	*B*. *suis* bv. 1	*B*. *suis* bv. 1	6		NEW_1
ZW045	45	Cattle	*B*. *suis* bv. 1	*B*. *suis* bv. 1	6		NEW_3
ZW046	46	Cattle	B. suis bv. 1	B. suis bv. 1	6		NEW_3
ZW047	47	Cattle	*B*. *suis* bv. 1	*B*. *suis* bv. 1	6		NEW_1
ZW048	48	unknown	*B*. *suis* bv. 1	*B*. *suis* bv. 1	6		NEW_1
ZW201	201	Pig	*B*. *suis* bv. 1	*B*. *suis* bv. 1	6		NEW_4
ZW053	53	Cattle	*B*. *abortus* bv. 1	ᵃ	28	82	NEW_5
ZW323	323	Cattle	*B*. *abortus* bv. 1	ᵃ	28	82	Temp820
ZW100	100	Dog	*B*. *canis*	*B*. *canis*	3	26	NEW_6
ZW377	377	Dog	*B*. *canis*	*B*. *canis*	3	26	NEW_7
ZW002	2	Sheep	*B*. *ovis*	ᵃ	ᵇ	ᵇ	ᵇ
ZW005	5	Sheep	*B*. *ovis*	ᵃ	ᵇ	ᵇ	ᵇ
ZW248	248	Cow	*B*. *abortus*	ᵃ	ᵇ	ᵇ	ᵇ
ZW283	283	Cow	*B*. *abortus*	ᵃ	ᵇ	ᵇ	ᵇ
BCCN R7	292	cattle	*B*. *abortus bv*. *4*	ᵃ	30	78	Temp834
BCCN R5	86/8/59	cattle	*B*. *abortus bv*. *2*	ᵃ	29	80	Temp836
BCCN R4	REF544	cattle	*B*. *abortus* bv. 1	ᵃ	30	78	Temp834
BCCN R18	REFRM 6/66	dog	*B*. *canis*	*B*. *canis*	3	26	Temp17
BCCN R17	REFBOW 63/290	sheep	*B*.*ovis*	ᵃ	1	25	Temp829
BCCN R12	REF1330	swine	*B*. *suis* bv 1	*B*. *suis* bv 1	6	33	Temp3

ᵃ Strains cannot be differentiated with the assay

ᵇ template DNA unavailable for further testing with MLVA

Bruce-ladder gave identical results (Fig 1B and Table 2) as AMOS-PCR and in addition could identify strains ZW100 and ZW377 as *B*. *canis*. Using the complementary Suis-ladder multiplex PCR [46], both strains were confirmed to be *B*. *canis* and strains ZW011, ZW40, ZW043, ZW045-048, ZW201 were confirmed as *B*. *suis* bv. 1 (**S1 Fig**).

### MLVA

Due to lack of sufficient DNA, strains ZW002 and ZW005 (both *B*. *ovis*), and ZW248 and ZW283 (both *B*. *abortus*) could not be genotyped using MLVA16. MLVA data derived from the seven reference strains were as expected from previously published data, with the exception of reference strains 16M, 63/290 and RM 6/66. The 16M strain used in the present study differs from the *B*. *melitensis* reference 16M strain at locus Bruce07, which is not unexpected due to high variability at this locus [25]. The reference strain RM 6/66 we used differed from *B*. *canis* reference RM 6/66 strain at Bruce07, Bruce09 and Bruce16 loci whereas 63/290 differs at loci Bruce09 and Bruce16. The difference between control strains used in this study and the reference strains may be due to amplification of the reference DNA using Genomiphi (GE Healthcare Life Sciences AEC-Amersham) due to low quantities of DNA from these strains available in our study.

**Fig 2:**
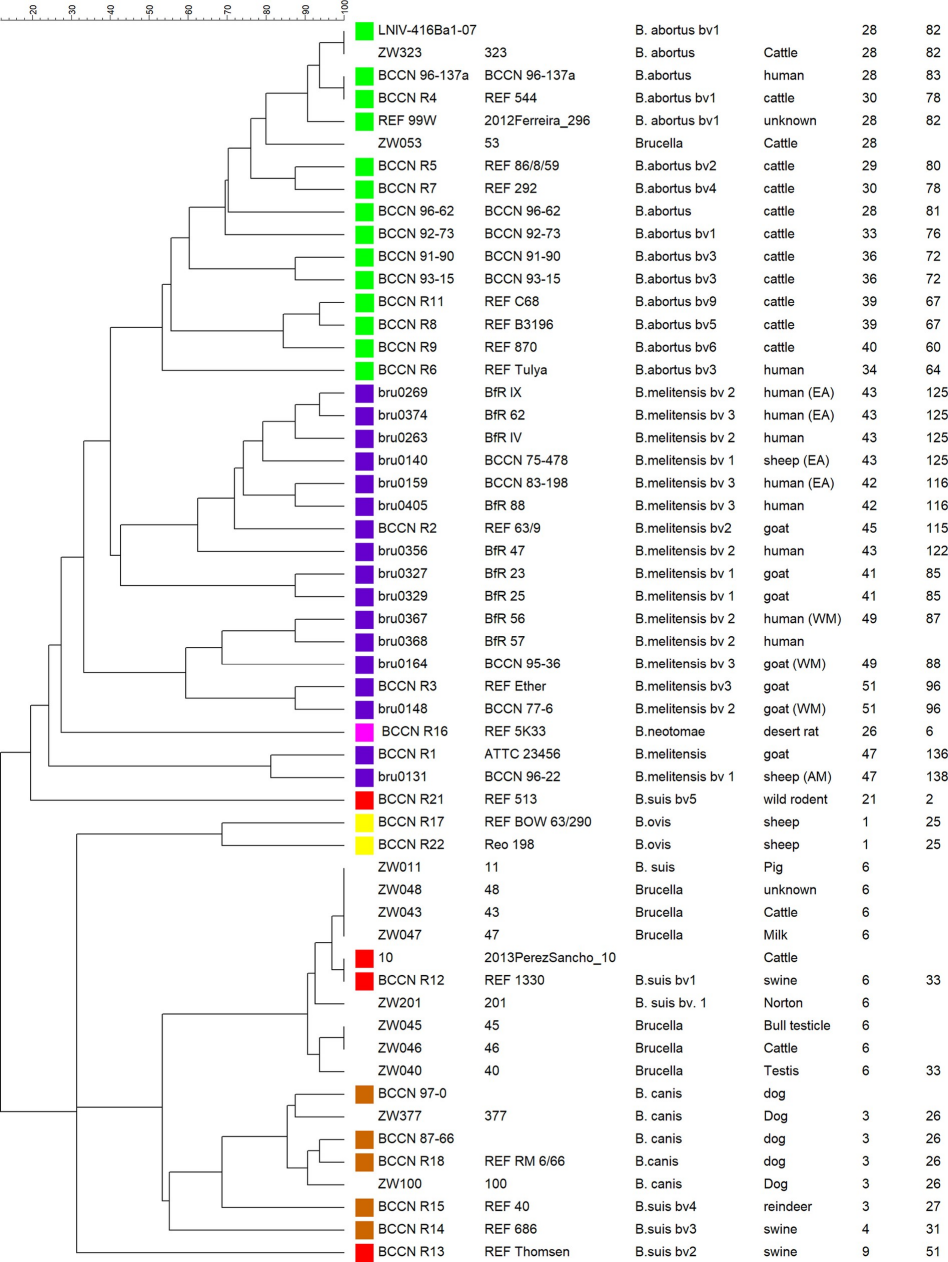
UPGMA algorithm cluster analysis of 54 *Brucella* strains (published data and Zimbabwean (ZW numbers)) using MLVA16. The dendrogram is based on 47 genotypes obtained from 54 strains. The color code reflects the grouping of *Brucella* species using minimal spanning tree of MLVA8 (S1 Fig) with white color-coded Zimbabwean *Brucella* strains, brown *B*. *canis* and *B*. *suis bv*. 3 and 4, red *B*. *suis* bv. 1, 2 and 5, yellow *B*. *ovis*, dark blue *B*. *melitensis*, green *B*. *abortus* and pink *B*. *neotomae*. The last three columns indicate MLVA8 (panel 1), MLVA11 (panel 1 & 2A) and MLVA16 (panel 1; 2A & 2B) genotype identification.

The Zimbabwean strains consisted of eight MLVA16 genotypes and clustered into three groups when analyzed together with MLVA data from [45]. All eight *B*. *suis* bv. 1 strains (ZW011, 040, 043, 045, 046, 047, 048 and 201) belong to MLVA8 genotype 6 like the vast majority of B. suis bv. 1 strains in the MLVA bank and are most closely related to *B*. *suis* bv. 1 reference strain 1330 in the *B*. *suis* bv. 1, 3, 4 / *B*. *canis* MLVA cluster (Fig 2). ZW100 and ZW377 (both isolated from dogs in Harare) formed a sub-cluster with *B*. *canis* REF RM 6/66 in the *B*. *suis* bv. 1, 3, 4 / *B*. *canis* cluster (Fig 2). *B*. *abortus s*train ZW323 (MLVA8 genotype 28) was identical at all 16 VNTR loci to *B*. *abortus* bv. 1 strain (LNIV-416Ba1-07) from Portugal [44] while *B*. *abortus* bv. 1 ZW053 strain also belonged to MLVA8 genotype 28. The clustering obtained with the Minimum Spanning Tree (MST) analysis is similar to the UPGMA clustering (S2 Fig).

### Whole genome sequence and WGS-SNP analysis

**Fig 3:**
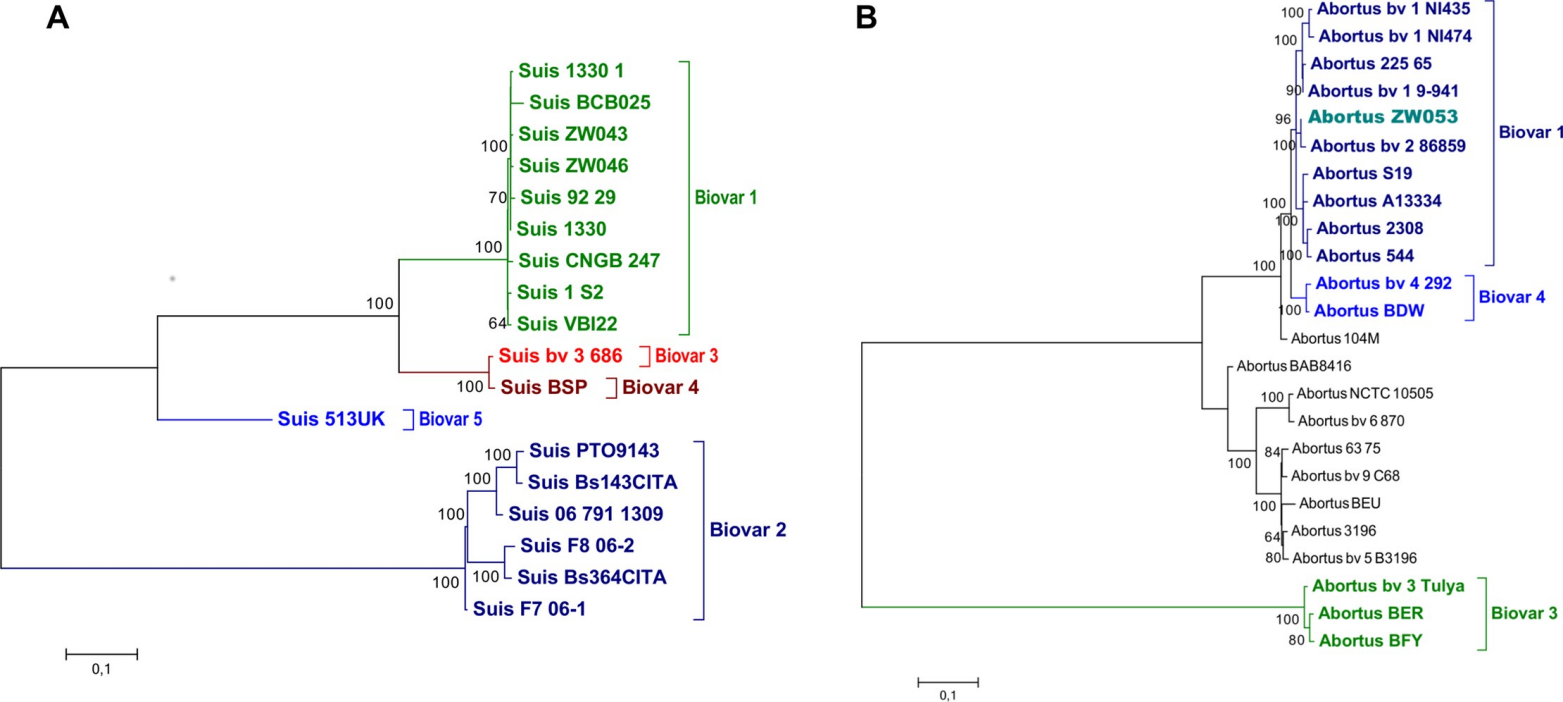
**Phylogenetic trees of (A) *Brucella suis* and (B) *B*. *abortus* using SNPs from whole genomes.** Dendrograms were generated using maximum likelihood with 500 bootstrap replicates, using 7104 and 4549 SNPs of *B*. *suis* and *B*. *abortus* genomes respectively.

WGS-SNP phylogenetic analysis of 19 *B*. *suis* and 24 *B*. *abortus* genomes was defined by 7104 and 4549 core SNPs respectively. Phylogenetic analysis of the *Brucella* genomes showed that *B*. *abortus* ZW053 clustered in *B*. *abortus* bv. 1 and 2 clade alongside *B*. *abortus* bv.2 86/8/59, while *B*. *suis* ZW043 and ZW046 strains are grouped within the *B*. *suis* bv. 1 clade (Fig 3). Comparative SNP analysis between ZW053 and *B*. *abortus* bv. 2 str. 86/8/59 resulted in 35 SNPs as compared to 90 SNPs obtained when comparing the strain with *B*. *abortus* bv.1 str 9–941.

## Discussion

Fast and accurate diagnosis of brucellosis is important for control programs [23] and since the choice of the assay to use depends on the affordability and availability of expertise in a given country, it is always a trade-off between the two requirements. Eradication and control program based on compulsory calf vaccination with *B*. *abortus* strain S19 was introduced in Zimbabwe in the early 1980s, but only to commercial farms and was voluntary to communal ones [17, 18]. However, infections caused by *B*. *abortus* and *B*. *melitensis* have been reported from both the communal and the commercial areas of Zimbabwe [13, 14, 15]. PCR-based assays can be used as a supplement or even a replacement to biotyping for the identification of *Brucella* species and/or biovars [23, 25], as genotyping is often essential for accurate epidemiological inference. Biotyping is time consuming, labour intensive and requires good expertise specific for this pathogen. In addition, it involves handling of live cultures that poses risks of laboratory exposure and infection [52]. The purpose of the study was to explore the practical suitability of PCR assays (MLVA, AMOS-PCR and Bruce-ladder) for laboratories that do not have biotyping capabilities as was the case with CVL, Zimbabwe at the time of the identification of these *Brucella* strains isolated from cattle, pigs, dogs and sheep. *B*. *abortus* and *B*. *melitensis* are the most prominent species in Africa and were previously reported in Zimbabwe from livestock and wildlife [13, 14]. The occurrence of these species in wildlife complicates the control of bovine brucellosis since it is almost impossible to vaccinate wildlife. Furthermore, interaction between wild life and animals in areas bordering the National parks could result in possible transmission of the disease.

In the present report, eight strains including five isolated from bovine and two from pigs were identified as *B*. *suis* bv. 1. The strains were identified as *B*. *suis* using AMOS-PCR [24, 25] and Bruce-ladder [26]. Suis-ladder [43] and MLVA identified these isolates to be *B*. *suis bv*. 1. Four strains isolated from cattle were identified as *B*. *abortus* bv.1 with AMOS-PCR and Bruce-ladder in this study. The identification could be confirmed by MLVA in two cases only due to limited DNA availability. In a previous study [13], *B*. *abortus* bv. 1 was shown to be the main cause of bovine brucellosis in Zimbabwe; however, in this study *B*. *suis* bv. 1 was most frequently isolated strain even from cattle. The isolation of *B*. *suis* bv.1 from both pigs and cattle might be the result of either mixed farming or the interaction of animal species in the grazing areas and drinking points. MLVA, Bruce-ladder and Suis-ladder assays identified two strains ZW100 and ZW377 as *B*. *canis*. This is the first report of *B*. *canis* in Zimbabwe. Due to low quantity of DNA, two *B*. *ovis* strains (ZW002 and ZW005) were only identified with AMOS and Bruce-ladder PCR but not with MLVA. *Brucella ovis* has been indicated by OIE reports as present in Zimbabwe [19].

Two *B*. *suis* bv. 1 strains isolated from cattle were selected for draft whole genome sequencing since *B*. *suis* had not been reported from pigs in Zimbabwe in literature but was detected in samples from both cattle and pigs in this study. WGS indicated that the two strains are separated from *B*. *suis* bv. 1 reference strain 1330 [37] by only five SNPs. A third strain, identified as *B*. *abortus* bv.1 was shown by WGS-SNP analysis to be closest to a strain independently recovered from Zimbabwe.

A previous study [24] compared the AMOS, Bruce-ladder and MLVA8 assays for typing of *Brucella* species and found only Bruce-ladder correctly identified all tested *Brucella* strains as MLVA8 does not resolve the very closely related *B*. *canis* and *B*. *suis* bv. 4. Both MLVA11 and MLVA16 resolve the two species however and also allows comparison to a worldwide *Brucella* MLVA dataset [53]. As shown in previous studies [39, 40, 41], WGS-SNP analysis provides better resolution than MLVA16, and much stronger phylogenetic support although there are still fewer strains from more limited geographic areas available for comparisons as compared to the MLVA database. Importantly the number of public whole genome sequences, particularly sequence reads archives, is rapidly growing with already more than 1000 datasets available.

The status of *B*. *suis* as a single species has been questioned in light of a broader host specificity [54]. Isolation of *B*. *suis* bv. 1 from bovines in Zimbabwe was first reported in 2014 [47]. The present study further emphasizes the occurrence of *B*. *suis* bv. 1 in cattle and pigs. There are several reports of isolation of *B*. *suis* bv. 1 from cattle [55, 56] in which the infection appears to be noncontagious with limited induced pathology and no induction of abortions [19, 54]. The presence of *B*. *suis* bv. 1 in pigs and bovines in Zimbabwe could be due to the predominance of smallholdings with mixed populations of livestock [57]. Therefore, the use of multiplex PCR assays that will distinguish the four species (*B*. *ovis*, *B*. *abortus*, *B*. *suis* and *B*. *canis*) present in Zimbabwe as confirmatory test will strengthen the control programs since most serology assays are based on smooth lipopolysaccharides (LPS) which cannot detect *B*. *ovis* and *B*. *canis* as they are rough strains.

WGS analysis showed that the ZW053 strain from a bovine in Zimbabwe [47] has large insertions and deletions as described in other *B*. *abortus* genomes [38, 58]. In spite of the variations observed in the genome sequences (S2 Table), whole-genome sequencing of the three strains and their comparison to reference genomes indicate that the isolates were *B*. *suis* (ZW043 and ZW046) and *B*. *abortus* (ZW053) respectively, thus corresponding with the data obtained with the Bruce-ladder, AMOS, Suis-ladder and MLVA PCR assays. Isolates from sub-Saharan countries and those from Europe have been shown to respectively cluster together, although heterogeneity within these species especially *B*. *abortus* do exist [12, 59]. This was also the case with ZW053 as it grouped with a Portuguese strain, and we hypothesize that this might be the result of socio-economic, migration or colonization links among Zimbabwe, Mozambique and Portugal or more generally European countries. Clustering of *B*. *abortus* bv. 2 strain 86/8/59 within biovar 1 and 2 clade and alongside ZW053 (Fig 3) and other *B*. *abortus* in WGS-SNP analysis was also shown in a previous study [41] that indicated that it might either be due to the paraphyletic nature of the biovar 1, 2 and 4 clade and the biovar classification not consistently reflecting genetic relationship in this species and/or that the biochemical biotyping to biovar level is unreliable. The present results further indicate the usefulness of MLVA and WGS-SNP in support of disease control. However, to perform the abovementioned assays requires a purified DNA template which may prove difficult to obtain due to the difficulty of culturing *Brucella*. Furthermore, brucellosis is endemic in sub-Saharan countries including Zimbabwe thus, the use of affordable high-throughput assays is necessary. More importantly, tests that can detect all the species that exit in a specific country should be considered.

Since most laboratories in Africa lack resources and expertise to do biotyping of *Brucella* to the species level, PCR assays like Bruce-ladder, AMOS and MLVA can contribute to the identification and can furthermore be used as an epidemiological tool and traceback of outbreaks. However, the choice of assays should be made considering reproducibility, robustness, expertise and affordability in a given setting and in most cases this choice will be a compromise. Brucellosis control programs in most countries are based on serological tests which includes Rose Bengal test (RBT), milk ring test, (MRT), complement fixation test (CFT), enzyme-linked immunosorbent assay (ELISA), the fluorescence polarisation assay (FPA) etc. [60]. These tests have varying sensitivity and specificity and they are prone to cross-reactions with other bacteria that have the smooth lipopolysaccharide used as the antigen in these assays [60]. Therefore, to complement these limitations, molecular assays can be used since most of them are robust, less expensive and can differentiate between *Brucella* spp. at genus, species and biovar levels [25, 26, 27, 28, 33, 44]. The development of standardised, safe and efficient DNA extraction procedures sufficient to produce a few micrograms of DNA of a good quality allowing long term conservation will be essential for this purpose.

Bruce-ladder and AMOS assays are species-specific simple and robust multiplex PCRs. Even though the initial AMOS PCR assay was more limiting as it has the capability of detecting only *B*. *abortus* bv 1, 2 and 4, *B*. *melitensis* bv. 1 and *B*. *suis* but not *B*. *canis*; it was subsequently enhanced and currently can detect *B*. *abortus* biovars 5, 6 and 9 and the new subgroup 3b of biovar 3 as well [61]. Furthermore, its subsequent use alongside Bruce-ladder is also an advantage. Moreover, a previous study [13] in which AMOS PCR assay was used, also indicated the presence of brucellosis in Zimbabwe with infections mainly caused by *B*. *abortus* bv. 1 (84.6%) and *B*. *abortus* bv. 2 (15.4%). The MLVA16 assay provides a clustering of strains that is in accordance with all currently recognized *Brucella* species and biovars [11, 32, 43].

Considering affordability and reproducibility; Bruce-ladder can be used as it allows identification of all known *Brucella* species including the vaccine strains simultaneously in one run. This study has confirmed that species differentiation can be correctly deduced from both MLVA16 and Bruce-ladder analysis. These PCR assays can therefore add to the control and eradication of brucellosis, since *B*. *ovis*, *B*. *abortus*, *B*. *suis* and *B*. *canis* could be identified. The latter two species are reported for the first time in Zimbabwe. Additionally, more strains, whole genome sequences, and epidemiological data from Zimbabwe are needed to accurately draw conclusions on the clustering and circulation of strains.

## Supporting information

S1 Table Reference strains and Zimbabwean *Brucella* spp. isolates identified by Bruce-ladder and repeat copy number of the indicated loci. (PDF)

S2 Table *Brucella abortus* and *B. suis* genome sequences retrieved from GenBank, used in the study for comparison of whole genome single nucleotide polymorphisms (WGS-SNPS) phylogenetic analysis. (PDF)

S1 Fig Suis-ladder multiplex PCR assay of *Brucella* DNA from Zimbabwe and reference strains. (TIF)

S2 Fig Minimum spanning tree analysis of published data and Zimbabwean *Brucella* isolates using the MLVA8 data (Panel 1 genotypes). (TIF)
