# Supplementary material for: Molecular characterization of Brucella species from Zimbabwe
Source: PLoS Negl Trop Dis. 2019 May 20;13(5):e0007311. doi: 10.1371/journal.pntd.0007311 (PMC6544324; doi:10.1371/journal.pntd.0007311)
Supplement: S1 Table — (PDF) [file pntd.0007311.s001.pdf]

Supporting information

S1 Table: Reference strains and Zimbabwean *Brucella* spp. isolates identified by Bruce-ladder and repeat copy number of the indicated loci

Strain number	Alternative number	Species	Host	Country[‡]	Bruce-ladder[†]	Genotype MLVA		Variable number of tandem repeat (VNTR) loci: Bruce															
								Panel 1								Panel 2A			Panel 2B				
						8	11	6	8	11	12	42	43	45	55	18	19	21	4	7	9	16	30
ZW011[*]	11	*B. suis*	Pig	Shamwa, ZW	*B. suis* bv. 1	6		2	3	6	10	4	1	5	2	4	40	9	6	6	5	5	3
ZW040	40	*Brucella*	Testis	Bindura, ZW	*B. suis* bv. 1	6	33	2	3	6	10	4	1	5	2	4	38	9	6	6	5	4	3
ZW043	43	*Brucella*	Cattle	Chiredzi, ZW	*B. suis* bv. 1	6		2	3	6	10	4	1	5	2	4	40	9	6	6	5	5	3
ZW045	45	*Brucella*	Bull testis	Bindura, ZW	*B. suis* bv. 1	6		2	3	6	10	4	1	5	2	4	40	9	6	6	5	4	3
ZW046	46	*Brucella*	Cattle	Norton, ZW	*B. suis* bv. 1	6		2	3	6	10	4	1	5	2	4	40	9	6	6	5	4	3
ZW047	47	*Brucella*	Milk	Zimbabwe	*B. suis* bv. 1	6		2	3	6	10	4	1	5	2	4	40	9	6	6	5	5	3
ZW048	48	*Brucella*	unknown	Zimbabwe	*B. suis* bv. 1	6		2	3	6	10	4	1	5	2	4	40	9	6	6	5	5	3
ZW201[*]	201	*Brucella*	Pig	Norton, ZW	*B. suis* bv. 1	6		2	3	6	10	3	1	5	2	4	40	9	6	6	5	5	3
ZW053	53	*Brucella*	Cattle	Matabeland, ZW	*B. abortus*	28	82	4	5	4	12	2	2	3	3	6	42	8	6	5	3	2	6
ZW323[*]	323	*B. abortus*	Cattle	Harare, ZW	*B. abortus*	28	82	4	5	4	12	2	2	3	3	6	42	8	3	5	3	4	5
ZW100[*]	100	*B. canis*	Dog	Harare, Highlands, ZW	*B. canis*	3	26	2	3	9	11	3	1	5	2	5	40	9	8	6	10	8	3
ZW377[*]	377	*B. canis*	Dog	Harare, ZW	*B. canis*	3	26	2	3	9	11	3	1	5	2	5	42	9	8	6	10	7	3
BCCN R7[#]	REF292	*B. abortus* bv 4	cattle	England	*B. abortus*	30	78	4	5	4	12	2	2	3	2	6	42	8	3	4	3	3	5
BCCN R6[#]	REFTulya	*B. abortus* bv 3	human	Uganda		34	64	3	5	4	11	2	2	3	3	8	40	8	6	5	3	11	5
BCCN R5[#]	REF86/8/59	*B. abortus* bv 2	cattle	England	*B. abortus*	29	80	4	5	4	12	2	1	3	3	6	42	8	3	4	3	3	5
BCCN R4[#]	REF544	*B. abortus* bv 1	cattle	England	*B. abortus*	30	78	4	5	4	12	2	2	3	3	5	42	8	3	5	3	4	5
BCCN R3[#]	REFEther	*B. melitensis* bv 3	goat	Italy		51	96	3	5	3	13	1	1	3	3	7	42	8	7	5	12	9	3
BCCN R22[#]	Reo 198	*B. ovis*	sheep	USA		1	25	3	5	3	10	1	1	5	2	3	7	9	9	9	9	15	2
BCCN R21[#]	REF513	*B. suis* bv 5	wild rodent	Former USSR		21	2	1	2	9	14	1	2	5	5	7	44	9	9	5	3	9	5
BCCN R2[#]	REF63/9	*B. melitensis* bv 2	goat	Turkey		45	115	1	5	3	12	2	2	3	2	4	40	8	7	4	3	5	4
BCCN R18[#]	REFRM 6/66	*B. canis*	dog	USA	*B. canis*	3	26	2	3	9	11	3	1	5	2	5	40	9	8	6	7	5	3
BCCN R17[#]	REFBOW 63/290	*B. ovis*	sheep	Australia		1	25	3	5	2	10	1	1	5	2	3	8	9	6	7	7	6	2
BCCN R15[#]	REF40	*B. suis* bv 4	reindeer	Former USSR		3	27	2	3	9	11	3	1	5	2	5	36	9	4	4	9	6	3

BCCN R14#	REF686	*B. suis* bv 3	swine	USA		4	31	2	3	4	11	3	1	5	2	4	38	9	7	7	10	4	5
BCCN R13#	REFThomsen	*B. suis* bv 2	swine	Denmark		9	51	2	4	8	15	6	1	5	2	6	44	9	9	9	18	2	4
BCCN R12#	REF1330	*B. suis* bv 1	swine	USA	*B. suis* bv 1	6	33	2	3	6	10	4	1	5	2	4	38	9	6	6	5	5	3
BCCN R11#	REFC68	*B. abortus* bv 9	cattle	England		39	67	3	5	3	12	2	2	2	3	7	42	8	6	6	3	3	3
BCCN R1#	ATTC 23456	*B. melitensis*	goat	USA		47	136	3	4	2	13	4	2	3	3	5	36	6	2	5	8	3	6
BCCN R9#	REF870	*B. abortus bv6*	cattle	Africa		40	66	3	5	3	12	2	2	3	3	7	42	8	3	6	3	3	3
BCCN R8#	REFB3196	*B. abortus bv5*	cattle	England		39	67	3	5	3	12	2	2	2	3	7	42	8	6	7	3	3	3
BCCN R16#	REF5K33	*B. neotomae*	desert rat	USA		26	6	4	5	4	13	4	3	5	1	6	42	9	3	3	3	6	3
ZW002	2	*Brucella*	Sheep	Gwanda, ZW	*B. ovis*			ND															
ZW005*	5	*B. ovis*	Sheep	Insiza, ZW	*B. ovis*			ND															
ZW248	248	*Brucella*	Cow	Mazowe, ZW	*B. abortus*			ND															
ZW283*	283	*B. abortus*	Cow	Gwanda, ZW	*B. abortus*			ND															

*Identified using growth characteristics and biochemical profiles excluding the phages lysis which was confirmed by PCR assays in this study.

‡ ZW indicate strains isolated from Zimbabwe and REF indicate reference DNA obtained from BCCN: Brucella culture collection Nouzilly.

† Isolates identified to species level using Bruce-ladder PCR assay and B. canis and B. suis further characterized using Suis-ladder PCR assay.

#MLVA data obtained from Maquart et al. (26).

ND Not determines due to low quantity of DNA.

MLVA8 = Panel 1; **MLVA 11** = Panels 1 & 2A; **MLVA16** = Panels 1, 2A & 2.
