# Supplementary material for: Molecular characterization of Brucella species from Zimbabwe
Source: PLoS Negl Trop Dis. 2019 May 20;13(5):e0007311. doi: 10.1371/journal.pntd.0007311 (PMC6544324; doi:10.1371/journal.pntd.0007311)
Supplement: S2 Table — (PDF) [file pntd.0007311.s002.pdf]

S2 Table: List of *B. abortus* and *B. suis* genome sequences retrieved from GenBank, used in the study for comparison of whole genome single nucleotide polymorphisms (WGS-SNPS) phylogenetic analysis.

**Organism**	**Accession no.**	**Sequenced year**
B. suis 1330	NC_017251, NC_017250	2002
B. suis 1330 (1)	CP002997, CP002998	2011
B. suis VBI22	NC_016797, NC_016775	2011
B. suis 513UK	CP007717, CP007716	2011
B. suis BSP	CP008757, CP008756	2014
B. suis ZW043	CP009094, CP009095	2014
B. suis ZW046	CP009096, CP009097	2014
B. suis bv. 2 PT09143	CP007691, CP007692	2014
B. suis bv. 2 Bs143CITA	CP007695, CP007696	2014
B. suis bv. 2 Bs364CITA	CP007697, CP007698	2014
B. suis bv. 3 str. 686	NZ_ACBL00000000	2009
B. suis 92/29	NZ_AQNU00000000	2013
B. suis CNGB 247	NZ_AQLA00000000	2013
B. suis F7/06-1	NZ_AQLD00000000	2013
B. suis F8/06-2	NZ_AQOA00000000	2013
B. suis S2-30	NZ_ALOS00000000	2012
B. suis 06 791 1309	AXMT01000001, AXMT01000011	2013
B. suis bv. 1 str. BCB025	NZ_ALOK00000000	2012
B. suis bv. 1 str. S2	NZ_AFFC00000000	2011
B. abortus bv 1 str. NI435a	AGVF00000000.1	2012
B. abortus bv 1 str. NI474	GCA_000245875.1	2012
B. abortus 225/65	GCA_000369905.1	2012
B. abortus bv. 1 str. 9-941	NC_006932.1, NC_006933.1	2005
B. abortus bv. 2 str. 86/8/59	NZ_CP007765.1, NZ_CP007764.1	Reference
B. abortus A13334	NC_016795.1, NC_016777.1	2012
B. abortus S19	NC_010742.1, NC_010740.1	2008
B. abortus 2308	AM040264, AM040265	2005
B. abortus bv. 6 str. 870	CP007709.1, CP007710.1	2014
B. abortus bv. 9 str. C68	CP007705.1, CP007706.1	2014
B. abortus str. NCTC 10505	CP007700.1, CP007701.1	2014
B. abortus str. BAB8416	CP008774.1, CP008775.1	2016
B. abortus str. 3196	CP007707.1, CP007708.1	2014
B. abortus bv.1 str. 544	JPHK00000000	2015
B. abortus bv.4 str. 292	JMSB01000000	2014
B. abortus str. BDW	CP007681.1, CP007680.1	2014
B. abortus str. 104M	ALOQ01000000	2012
B. abortus str. BEU	JMSA00000000	2014
B. abortus str. Tulya	ACBI01000000	2014
B. abortus str. BER	CP007682, CP007683	2014
B. abortus str. BFY	CP007738, CP007737	2014
B. abortus ZW053	CP009098.1, CP009099.1	2014
B. abortus str. 6375	CP007663, CP007662	2014
B. abortus bv. 5 str. B3196	ACXC01000000	2010
