# Supplementary figures and images for: Molecular characterization of Brucella species from Zimbabwe
Source: PLoS Negl Trop Dis. 2019 May 20;13(5):e0007311. doi: 10.1371/journal.pntd.0007311 (PMC6544324; doi:10.1371/journal.pntd.0007311)

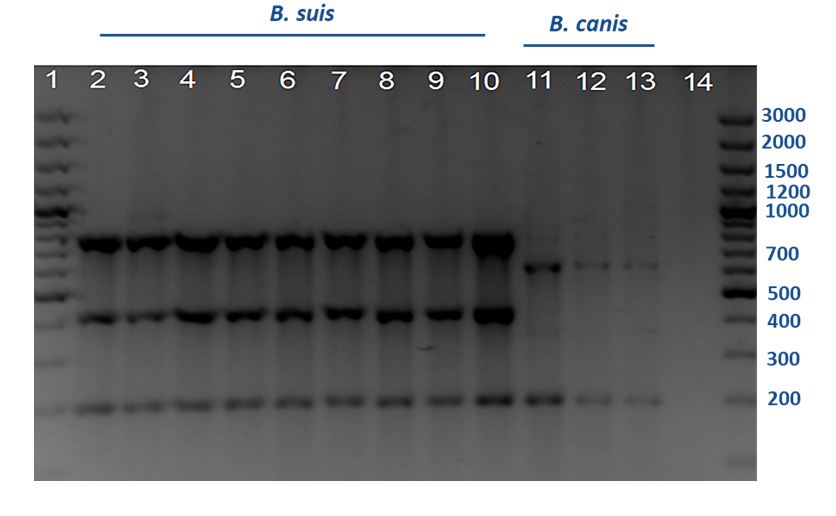

Supplement: S1 Fig — (TIF) [file pntd.0007311.s003.tif]

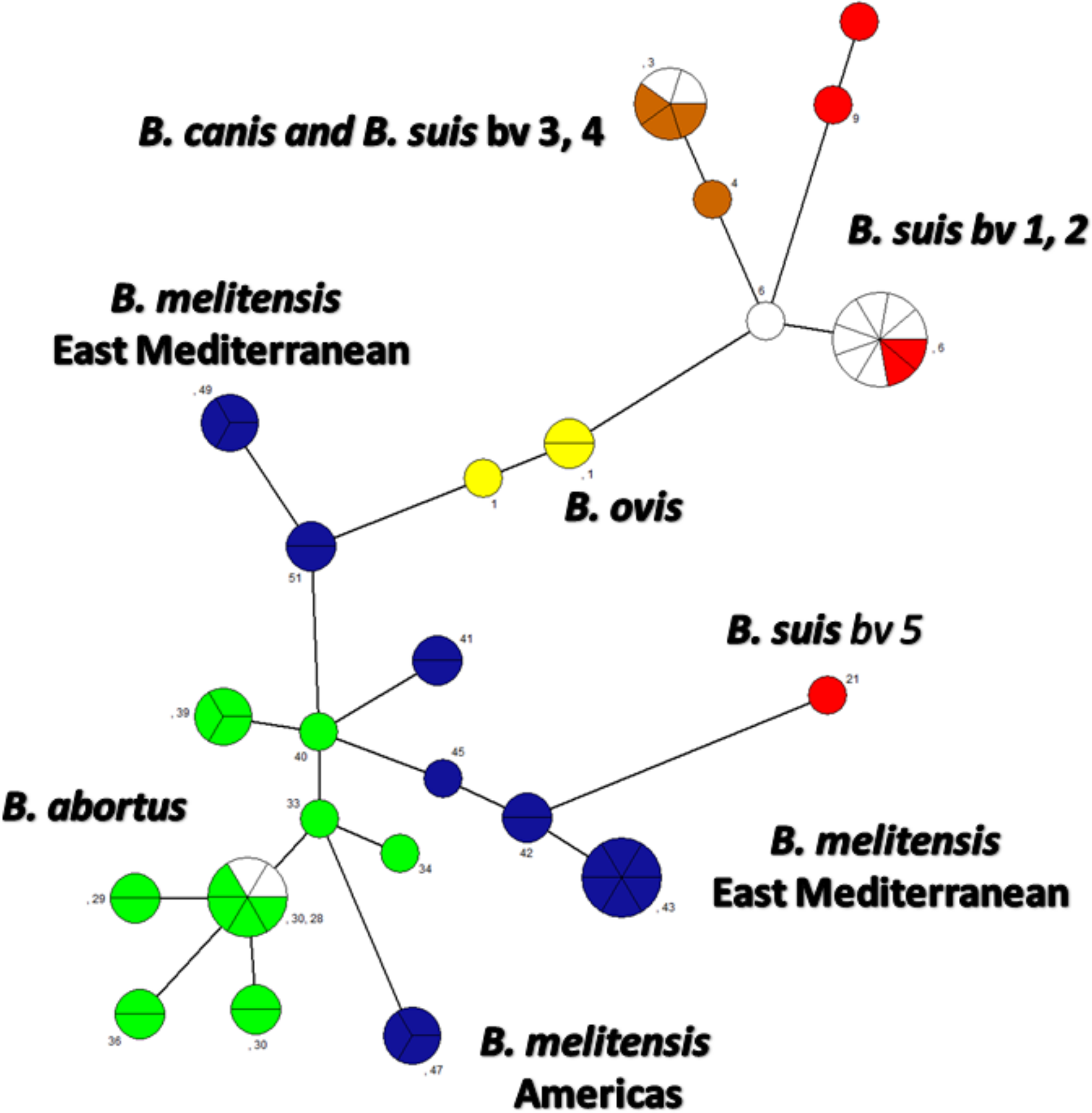

Supplement: S2 Fig — (TIF) [file pntd.0007311.s004.tif]
